# Editorial: Recent advances in diagnosis and treatment of comorbid conditions in eating disorders

**DOI:** 10.3389/fpsyt.2022.1117831

**Published:** 2023-01-24

**Authors:** Georgios Paslakis, Blake Woodside, Debra K. Katzman

**Affiliations:** ^1^University Clinic for Psychosomatic Medicine and Psychotherapy, Medical Faculty, Ruhr-University Bochum, Luebbecke, Germany; ^2^Centre for Mental Health, University Health Network, Toronto, ON, Canada; ^3^Division of Adolescent Medicine, Department of Pediatrics, The Hospital for Sick Children and University of Toronto, Toronto and the Research Institute, The Hospital for Sick Children, Toronto, ON, Canada

**Keywords:** eating disorder, comorbidity, treatment, psychotherapy, anorexia nervosa, bulimia nervosa, binge eating disorder

Individuals with eating disorder (ED) often experience both an ED and at least one other psychiatric and/or medical disorder. Such comorbidity contributes to the ED severity and poor treatment outcomes. In the present Special Issue dedicated to “*Recent Advances in Diagnosis and Treatment of Comorbid Conditions in Eating Disorders*,” researchers in the field of EDs have shared their most recent work regarding the impact of medical and psychiatric comorbidities on diagnosis, course of treatment, and treatment outcomes in individuals with EDs. The present issue synthesizes the current evidence base and identifies gaps in research and care. It covers recent literature relating to the psychiatric and medical comorbidities of feeding and EDs, some of which precede the ED, occur alongside, or are a consequence of the ED.

The study by Norris et al. assessed specific traits, comorbidities, and treatment requirements in a cohort of adolescents with avoidant restrictive food intake disorder (ARFID) in a specialized clinic in Ottawa, Canada. The researchers found high rates of psychiatric comorbidity, mostly anxiety, followed by mood disorders, attention deficit hyperactivity disorder, autism spectrum disorder, obsessive-compulsive disorder (OCD), and learning difficulties. The study highlights the clinical complexity observed in youth with ARFID, and the need for specialized services for these young people.

The study by Devoe et al. presents a large systematic review and meta-analysis that includes 35 studies and a total of 9,646 individuals with a clinically diagnosed ED, and examined the prevalence rates of impulse control disorders and behavioral addictions. The authors found a 22% prevalence for any impulse control disorder, especially for pathological/compulsive buying, predominantly in individuals with EDs of the binge-purge phenotype. This study supports the need for screening and monitoring of comorbid impulse control disorders and behavioral addictions in individuals with EDs.

Riquin et al. investigated psychiatric comorbidities in adults with anorexia nervosa (AN) and found that more than half of the cohort had experienced major depressive disorder (MDD) over their lifetime, almost a third had had generalized anxiety disorder (GAD) or social phobia (SP), and more than a quarter had OCD. Interestingly, the study showed that individuals with AN had more severe symptoms when comorbid MDD and GAD were diagnosed prior the diagnosis of the AN. The authors also reported that the greatest clinical severity (i.e., ED-related symptoms), general clinical condition, and quality of life in individuals with AN was associated with lifetime comorbidity for MDD, GAD, or SP.

Li et al. evaluated treatment outcomes based on self-reported assessment of autistic traits -high autistic traits (HAT) vs. low autistic traits (LAT)- in individuals with AN in either an inpatient or day treatment program. In individuals with AN, HAT was a positive predictor for clinical improvement of the ED symptoms in an inpatient treatment program, but a negative predictor for those in a day treatment program. The authors argued that the individualized and structured routine care in an inpatient setting may be more suitable for the treatment of individuals with comorbid AN and HAT. While research in this area is growing, these findings suggest that treatment adaptations for individuals with comorbid AN and autistic traits are needed.

Nobile et al. examined cognitive functions including attention, decision making, set shifting, and central coherence in women with bulimia nervosa (BN) who were receiving hormonal contraceptives (HC) compared to those who were not. The authors adjusted their analyses for a variety of variables that might influence cognitive performance and found that women with BN on HC (all types) showed better selection abilities and global sustained attention, and better understood the rules of the Iowa gambling task for decision making, while no differences between the groups were found for set shifting and central coherence. The authors argued that by limiting sex hormone changes by means of HC, cognitive functions might appear less prone to fluctuations.

Finally, Mazurak et al. examined sleep duration and other sleep-associated outcomes in higher weight children and adolescents undergoing an inpatient intervention for weight loss compared to a control group. Parents reported significantly poorer sleep outcomes in the group of higher weight children and adolescents compared to the control group, while at the same time, inpatient treatment had no effect on these outcomes. The authors discuss the need to address sleep behaviors as part of weight-loss therapy as well as the necessity for further research on the inferences between sleep outcomes and further psychological factors in children's cohorts.

This Special Issue highlights recent literature on the implications of psychiatric and medical comorbidities reported in individuals with EDs. Individuals with EDs have a higher mortality rate compared to the general population and death is often due to the medical and/or psychiatric comorbidity -suicide or severe medical complications. The studies in the issue illustrate that comorbidities, some that precede the ED and others that are the consequence of the ED, can impact the clinical presentation, diagnosis, and treatment. Further, the comorbidity associated with EDs makes treatment a challenging task. This issue adds to the existing knowledge and provides stimuli to improve early diagnosis and treatment interventions to reduce the burden of suffering in individuals with EDs and comorbid conditions.

## Author contributions

All authors contributed to writing and editing. All authors contributed to the article and approved the submitted version.

